# Impact of Anesthetic Agents on Endothelial Glycocalyx Injury during Total Knee Arthroplasty: Desflurane- vs. Propofol-Based Anesthesia—A Prospective Randomized Controlled Trial

**DOI:** 10.1155/2021/8880267

**Published:** 2021-01-23

**Authors:** Chung-Sik Oh, Ji Min Choi, Eun Hi Park, Liyun Piao, Hyun-Jun Park, Ka-Young Rhee, Seong-Hyop Kim

**Affiliations:** ^1^Department of Anesthesiology and Pain medicine, Konkuk University Medical Center, Konkuk University School of Medicine, Seoul, Republic of Korea; ^2^Rsearch Institute of Medical Science, Konkuk University School of Medicine, Seoul, Republic of Korea; ^3^Department of Infection and Immunology, Konkuk University School of Medicine, Seoul, Republic of Korea; ^4^Korea Institute of Radiological & Medical Sciences, Seoul, Republic of Korea

## Abstract

**Background:**

Ischemia-reperfusion injury and inflammation after tourniquet deflation in total knee arthroplasty are known to be associated with endothelial glycocalyx (EG) injury. This study is aimed at comparing EG injury between desflurane- and propofol-based anesthesia in patients undergoing total knee arthroplasty.

**Materials and Methods:**

Patients were allocated to the desflurane group or propofol group. The opioid remifentanil was administered intraoperatively in both groups. Blood samples were obtained from the arterial line preoperatively, immediately before and 5 min after tourniquet deflation, and at 1, 6, and 24 h, postoperatively. Serum syndecan-1, cytokines (interleukin-1*β*, 6, 10, and tumour necrosis factor-*α*), and other laboratory values were investigated.

**Results:**

Eighty patients were included in the final analysis. The change in syndecan-1 did not significantly differ between the desflurane and propofol groups (peak median level of syndecan-1; 754.5 pg/ml vs. 780.3 pg/ml, respectively, *P* = 0.512). Laboratory values (serum cytokines, creatinine phosphokinase, lactate dehydrogenase, and lactate levels) were also similar between the two groups. Pulmonary oxygenation was briefly improved after tourniquet deflation in the desflurane group but was similar between the two groups begging at 1 h, postoperatively.

**Conclusions:**

The effect of desflurane was not superior to that of propofol in protecting the EG from ischemia-reperfusion injury during total knee arthroplasty. This trial is registered with Trial Registry Number NCT02756715 (http://clinicaltrials.gov).

## 1. Introduction

Total knee arthroplasty (TKA) is one of the most commonly performed orthopedic procedures in elderly patients [1, 2]. For most patients, a tourniquet is applied during surgery to minimize surgical bleeding [3]. Ischemia-reperfusion injury (IRI) can occur after tourniquet release following TKA and has been shown to result in skeletal muscle complications [4, 5]. In addition to muscle injury, IRI induces inflammation and related complications that affect morbidity and mortality after TKA [6].

The endothelium regulates vascular homeostasis. The EG, which covers the luminal endothelium, plays a crucial role in regulating the physiologic functions of the endothelium [7]. Multiple studies have shown that trauma, sepsis, IRI, and other factors can induce EG injury [8, 9]. In particular, the occurrence of IRI is a critical factor in the induction of EG injury [10]. Therefore, efforts should be made to minimize IRI after tourniquet release during TKA. Volatile anesthetic agents are known to exhibit organ protective effects against IRI [11], and the preconditioning effect of volatile anesthetics plays a key role in organ protection by reducing inflammation and oxidative stress [12, 13]. Volatile anesthetics attenuated IRI-related endothelial destruction in some animal studies [13–15]. However, clinical studies investigating the endothelial protective effect of volatile anesthetics show conflicting results [16, 17]. In addition, few clinical studies revealed the different effect of volatile anesthetics and propofol in terms of endothelial protection against IRI during TKA.

Therefore, we hypothesized that volatile anesthetic-based anesthesia would be associated with reduced EG injury, compared with intravenous anesthetic-based anesthesia, in patients undergoing TKA, using a tourniquet. This study investigated the changes in serum levels of syndecan-1 between desflurane- and propofol-based anesthesia after tourniquet deflation during TKA.

## 2. Materials and Methods

### 2.1. Study Population

Ethical approval for this study (Approval number: KUH1160100) was provided by Institutional Review board of Konkuk University Medical Center, Seoul, Korea (Chairperson, Prof SH. Lee), on 24 March 2016 and registered at Clinical Research Information Service, United States National Library of Medicine (NLM) at the National Institutes of Health (NIH) (Registration number: NCT02756715; http://clinicaltrials.gov). This prospective, double-blind, randomized study was conducted at a single tertiary medical center (Konkuk University Medical Center, Seoul, Korea). The study was conducted from May 2016 to March 2018 in accordance with the original protocol, using a prospective randomized design. Written informed consent to participate was obtained from all patients. Only patients undergoing TKA were enrolled in the study. The exclusion criteria were as follows: (1) underlying neurological disorder, (2) underlying peripheral vascular disease, and (3) other concurrent surgery.

Before induction of anesthesia, patients were randomly allocated to receive desflurane-based anesthesia (desflurane group) or propofol-based anesthesia (propofol group) by opening sequentially numbered envelopes containing the randomisation assignment (third party allocation). Using random-permuted block randomisation, the allocation sequence was generated by the clinical research coordination center in our hospital, which was not otherwise involved in the trial. The anesthesia team and care providers including the surgical and nursing teams were blinded to the study goals; they were asked to strictly follow the study protocol. A research assistant was responsible for confirming patient eligibility and enrolling patients, including the acquisition of informed consent. A data collector was responsible for data gathering including blood sampling, and this collector was blinded to the group allocation. The research assistant and data collector did not participate in patient care. The research team members were blinded to the group allocation throughout the perioperative period and until completion of the statistical analysis.

### 2.2. Anesthetic Regimen

The anesthetic technique was standardized. All patients arrived at the operating room without premedication. Anesthesia was induced after establishment of routine patient monitoring pulse oximetry, electrocardiography, noninvasive blood pressure monitoring, near infrared spectroscopy, bispectral index (BIS), and radial artery cannulation on the contralateral side (relative to the surgical site) for invasive systemic arterial blood pressure monitoring. The anesthesiologists, who were blind to the study, were requested to anesthetise patients as outlined below. Lidocaine (0.5 mg/kg) was administered intravenously to patients in both groups. The desflurane group received thiopental sodium (5 mg/kg) intravenously to induce anesthesia. In the propofol group, an initial target concentration of 3.0 *μ*g/ml propofol (effect-site-modified Marsh model with a *k*_*e*0_ of 1.21/min) [18] was administered intravenously using a target-controlled infusion (TCI) device (Orchestra® Base Primea, Fresenius Vial, Brezins, France). After loss of consciousness, adequate mask ventilation was confirmed, and rocuronium (0.6 mg/kg) was administered intravenously for muscle relaxation. The fixed target concentration of remifentanil (5.0 ng/ml, plasma-site, Minto model) [19, 20] was administered intravenously and maintained until the end of surgery. After establishment of tracheal intubation, patients were ventilated with 40% oxygen in air, and the tidal volume was 6 ml/kg, based on lean body mass. Anesthesia was maintained with desflurane inhalation and with TCI of propofol in the respective groups by means of titrating BIS values between 40 and 60. Maximal and minimal end-expiratory concentrations of desflurane were recorded during anesthesia in the desflurane group; maximal and minimal effect-site target concentrations of propofol were recorded, during anesthesia in the propofol group. At the end of surgery, the administration of desflurane or propofol with remifentanil for each group was stopped. Residual neuromuscular paralysis was antagonised with sugammadex (2 mg/kg), and all patients were transferred to the postanesthesia care unit (PACU) after tracheal extubation. Newly prepared balanced crystalloid solution (Plasma Solution-A, HK inno.N, Seoul, Korea) was started at preoperative time. Crystalloid administration was maintained at a rate of 1 to 1.5 ml/kg/h during the intra- and postoperative period, until oral intake was restored in both groups. The loading of crystalloid solution was not performed during study period and hemodynamic stability after tourniquet release was maintained by using appropriate vasopressor. No colloid solution was used in the present study. Transfusion with packed red blood cells (pRBCs) was performed when the haematocrit (Hct) was <30% during the perioperative period in both groups.

### 2.3. Tourniquet

A 34-inch tourniquet (ZIMMER ATS 2000®, Zimmer Orthopedic Surgical Product, Dover, OH, USA) was applied to the thigh. An inflation pressure of 300 mmHg was applied immediately before the skin incision on the knee and maintained until dressing of the skin without interim release of the tourniquet. To prevent abrupt hypotension during tourniquet deflation, the tourniquet was released gradually over a period of 3 min after skin dressing in both groups, with use of vasoactive agents such as phenylephrine or ephedrine when necessary.

### 2.4. Blood Sampling

Blood was sampled immediately upon arrival, in ethylene-diamine-tetraacetic acid (EDTA) tubes. Blood samples were obtained without or with minimal stasis (within 30 sec) and immediately centrifuged at a rate of 1000 g for 15 min at 4°C. Serum samples were frozen within 1 h of sampling and stored at –80°C until analyzed.

### 2.5. Measurements

Syndecan-1 levels were measured as an indicator of EG injury. Serum cytokines including interleukin (IL)-1*β*, 6, 10, and tumor necrosis factor- (TNF-) *α* were measured to monitor inflammation. Syndecan-1 and cytokines were measured by enzyme-linked immunosorbent assay (ELISA) (triplicate measurements) with specific monoclonal antibodies using commercial EILSA kits (Quantikine® Colorimetric Sandwich ELISA kits (detection range from 10 to 10,000 pg/ml), R&D Systems, MN, USA). Blood samples were obtained before induction of anesthesia (Preop), immediately before tourniquet deflation (Before TQ), at 5 min after tourniquet deflation (After TQ), at discharge from the PACU (Post 1 h), at 6 h after discharge from the PACU (Post 6 h) and at 24 h after discharge from the PACU (Post 24 h). Serum creatinine (Cr), creatinine phosphokinase (CPK), and lactate dehydrogenase (LDH) were measured to check for muscle injury at Preop, Post 6 h, and Post 24 h. The ratio of arterial oxygen partial pressure to fractional inspired oxygen (PF ratio), Hct, and lactate were also evaluated at Preop, Before TQ, After TQ, and Post 1 h. Total amounts of intra- and postoperative transfusion of pRBCs were recorded; the amount of postoperative bleeding was measured over the following 24 h using an evacuator and collecting bag for wound drainage (Hemovac® evacuator, Zimmer Biomet, Warsaw, IN, USA).

### 2.6. Statistics

The primary outcome was the difference in syndecan-1 between desflurane and propofol groups. *A priori* power analysis yielded an effect size of 0.322 from our pilot study of 14 patients undergoing TKA. The calculated sample size for the primary outcome was 39 patients per group with an *α* value of 0.05 and power of 0.8. Therefore, we screened 43 patients in each group and a total of 86 patients were screened for eligibility in our study with a drop rate of 10%.

For continuous variables, the distribution of the data was first evaluated for normality using the Shapiro-Wilk test. Independent two-tailed *t*-tests were used to compare the means of continuous variables with normal distributions. The Mann–Whitney *U* test was used to compare variables that exhibited nonnormal distributions. Intragroup changes and intergroup differences in syndecan-1 levels over time were analyzed using repeated measurement analysis of variance. When significant differences were noted, Student's *t*-test or the Mann–Whitney rank-sum test (with Bonferroni's correction) was used to compare group differences. The chi-square test was used to compare categorical variables between the two groups. Normally distributed continuous data are presented as means ± standard deviations, and continuous data with nonnormal distributions are presented as medians (interquartile ranges). For categorical variables, the numbers of patients (*n*) and proportion (%) were calculated. All calculations were performed using Statistical Package for the Social Sciences 20® (IBM SPSS Inc., Chicago, IL, USA). Differences with *P* < 0.05 were considered statistically significant.

## 3. Results

**Figure 1:**
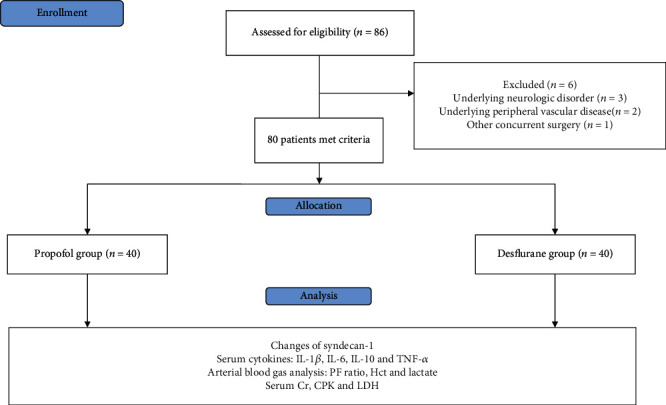
CONSORT flow diagram for the study. PF ratio: the ratio of arterial oxygen partial pressure to fractional inspired oxygen; Cr: serum creatinine; CPK: creatinine phosphokinase; LDH: lactate dehydrogenase; IL: interleukin; TNF: tumor necrosis factor.

In total, 86 patients were screened for eligibility in the study. Six patients were excluded for the following reasons: underlying neurological disorder (*n* = 3), underlying peripheral vascular disease (*n* = 2), and other concurrent surgery (*n* = 1). Therefore, 80 patients were enrolled and included in the final analysis and were assigned for 40 patients in each groups (Figure 1).

Patient characteristics were similar between the two groups with the exception of the amount of anesthetic agents administered, including sodium thiopental, desflurane, and propofol (Table 1).

**Figure 2:**
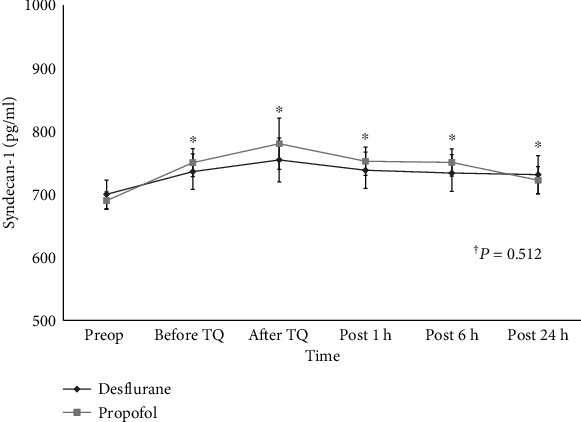
Changes in syndecan-1 level. ^†^*P* value indicate overall intergroup differences throughout all measured time points. ^∗^*P* < 0.05 in comparison with preoperative levels. Preop: before anesthesia induction; Before TQ: just before tourniquet deflation; After TQ: at 5 min after tourniquet deflation; Post 1 h: at discharge from postanesthesia care unit; Post 6 h: at 6 h after discharge from postanesthesia care unit; Post 24 h: at 24 h after discharge from postanesthesia care unit.

As TKA was performed, the levels of syndecan-1 increased, reaching a peak at 5 min after tourniquet deflation in both groups (*P* < 0.001). However, overall syndecan-1 levels did not significantly differ between the two groups throughout all measured time points (*P* = 0.512) and also did not show significant within group interactions according to time change within 24 h, postoperatively (*P* = 0.439) (Figure 2).

**Figure 3:**
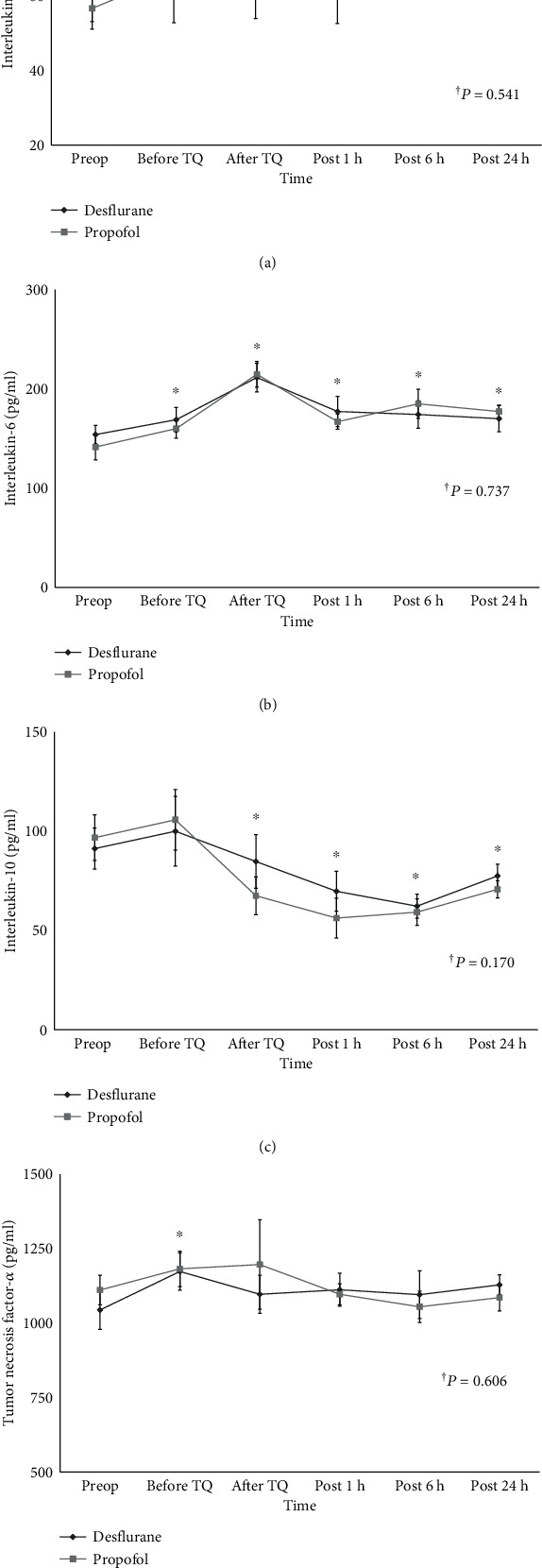
Changes in cytokine levels. (a) Interleukin- (IL-) 1*β*, (b) IL-6, (c) IL-10, and (d) TNF-*α*. ^†^*P* value indicate overall intergroup differences throughout all measured time points. ^∗^*P* < 0.05 in comparison with preoperative levels. Preop: before anesthesia induction; Before TQ: just before tourniquet deflation; After TQ: at 5 min after tourniquet deflation; Post 1 h: at discharge from postanesthesia care unit; Post 6 h: at 6 h after discharge from postanesthesia care unit; Post 24 h: at 24 h after discharge from postanesthesia care unit.

The overall changes in IL-1*β*, 6, 10, and TNF-*α* did not significantly differ between the two groups throughout all measured time points (*P* = 0.541, *P* = 0.737, *P* = 0.170, *P* = 0.606, respectively) (Figures 3(a)–3(d)). The changes in IL-1*β*, 6, 10, and TNF-*α* also did not show significant within group interactions according to time change within 24 h, postoperatively (*P* = 0.449, *P* = 0.107, *P* = 0.129, *P* = 0.142, respectively) (Figures 3(a)–3(d)).

Levels of serum Cr, CPK, and LDH did not significantly differ between the two groups at any time point examined (Table 2).

The PF ratio was significantly higher in the desflurane group in the propofol group at 5 min after tourniquet deflation (418 (391-481) in desflurane group vs. 389 (225-459) in propofol group; *P* = 0.009) (Table 3). However, the PF ratio did not significantly differ between the two groups at any other time point. The serum haematocrit and lactate levels were similar between the two groups at all time points examined (Table 3).

## 4. Discussion

In the present study, the inflammation and associated EG injury after tourniquet deflation during TKA did not significantly differ between the desflurane and propofol anesthesia groups. Although oxygenation was slightly better in the desflurane group immediately after tourniquet deflation during TKA, this improvement was not maintained at later time points.

The results of recent studies have suggested several strategies to minimize EG injury in clinical practice, such as maintenance of adequate organ perfusion, adjustment of coagulopathy to reduce bleeding, avoidance of fluid overloading, and other several strategies [8, 9]. The key method to minimize EG injury in the above strategies was the reduction of inflammation. In addition, several researchers showed that the choice of anesthetic agent could influence EG injury [12, 15, 21, 22]. Annecke et al. reported that compared to propofol-based anesthesia, the use of volatile anesthetic agents reduced EG injury in an animal IRI model [21]. Lin et al. also showed that propofol overdose caused EG injury in an animal model [22]. Casanova et al. suggested that the preconditioning effect of volatile anesthetic agents may reduce inflammation and therefore protect the EG [12]. However, the level of syndecan-1, which is a reliable marker of EG injury [23], did not significantly differ between desflurane and propofol anesthesia groups during TKA in the present study. Similarities in the levels of cytokines between the two groups suggested that volatile anesthetic agents and propofol have similar effects on inflammation during TKA. Levels of CPK and LDH, lactate, and postoperative bleeding were also similar between the two groups in the present study. Therefore, muscle injury due to mechanical stress during TKA is presumably unaffected by the type of anesthesia used. There are several possible reasons for this discrepancy. The intensity of ischemia may influence the preconditioning effect of volatile anesthetic agents. The protective effect of volatile anesthetics might be difficult to be demonstrated when the harmful stimuli such as ischemia is mild. Although TKA is not considered a minor surgery, intraoperative ischemia and inflammation are usually considered less severe in patients undergoing TKA than in patients undergoing other extreme conditions. Previous studies showed extreme conditions such as cardiac surgery, hemorrhagic shock, trauma, or sepsis have strong relationship with EG injury [10, 24, 25]. For instance, cardiopulmonary bypass, tissue hypoperfusion, coagulopathy, hemodilution, and various other possible factors in cardiac surgery may influence the occurrence of inflammation and related EG injury [10, 26]. Nevertheless, Landoni et al. recently showed both volatile anesthetics and propofol did not have superiority in terms of mortality even in cardiac surgery [27]. All patients in the present study were protected from such extreme conditions during TKA because we tried to maintain hemodynamic stability by using vasopressor, to prevent hypovolemic shock by using adequate tourniquet applying, to control infection by using antibiotics, and so on. Therefore, the EG injury might not appear strongly and the preconditioning effect of volatile anesthetics might not be expressed dramatically in the present study. Rehm et al. reported that the extent of EG injury could differ according to the severity of ischemia [28]. Majerczak et al. also reported that EG injury did not occur in healthy patients, even in the context of inflammatory conditions [29]. These results suggest a causal relationship between the severity of inflammation after IRI and the extent of EG injury. The duration of tourniquet application also could influence ischemia intensity. Olivecrona et al. reported that tourniquet application for a maximum of 120 min did not elicit ischemia-related complications during TKA [30]. Multiple other studies also suggested that the preconditioning effect of volatile anesthetic agents was ineffective in specific clinical conditions [17, 31]. Therefore, although volatile anesthetic agents are known to have a superior effect for EG protection some previous studies, this effect does not appear to be reproducible in clinical practice such as TKA.

The improvement of pulmonary oxygenation with desflurane-based anesthesia was observed in the present study. However, this effect seemed to be temporary. The level of oxygenation gradually became similar to the level observed in the propofol group, and the levels of different PF ratio between the two groups have little clinical relevance in the present study. An organ protective effect of volatile anesthetic agents was observed in the early phase immediately after the ischemic insult, but disappears within 2-4 h in previous studies [32, 33]. Therefore, the superiority of volatile anesthetic agents in terms of oxygenation remains uncertain in clinical practice. Additional studies are needed to investigate the effects of volatile anesthetics on pulmonary oxygenation after IRI.

The present study had several limitations. First, the study did not involve direct investigation of EG by electron microscopy. However, prior studies have shown that syndecan-1 is a reliable marker of EG injury and that its level is closely correlated with EG thickness and microvascular permeability [34, 35]. Second, blood samples were not obtained from the distal part of the surgical site to which the tourniquet was applied. Notably, samples were collected from the arterial line because of the difficult approach during surgery. There may be an extreme ischemic insult in venous blood at the distal part of the surgical site after tourniquet application. Further studies are needed to investigate direct EG injury during TKA.

## 5. Conclusions

In conclusion, the preconditioning effect of volatile anesthetic agents did not influence EG injury. Therefore, the specific anesthetic agent chosen for general anesthesia in patients undergoing TKA, involving the use of a tourniquet, may not provide a protective effect for the endothelium.

## Figures and Tables

**Table 1:** Demographic data.

		Desflurane group (*N* = 40)	Propofol group (*N* = 40)	*P*
Gender				1.000
	Male	5 (12.5%)	6 (15.0%)	
	Female	35 (87.5%)	34 (85.0%)	
Age (years)		73 (69-78)	72 (69-78)	0.870
Height (cm)		153.9 ± 7.7	153.6 ± 6.6	0.829
Weight (kg)		65.9 (56.2-71.7)	62.5 (54.1-74.0)	0.810
Surgical site				1.000
	Right	19 (47.5%)	19 (47.5%)	
	Left	21 (52.5%)	21 (52.5%)	
Underlying disease				
	HTN	31 (77.5%)	30 (75.0%)	1.000
	DM	8 (20.0%)	8 (20.0%)	1.000
	Coronary disease	4 (10.0%)	3 (7.5%)	1.000
Medication				
	Aspirin	4 (10.0%)	5 (12.5%)	1.000
	Clopidogrel	13 (32.5%)	14 (35.0%)	1.000
	NOAC	0 (0.0%)	3 (7.5%)	0.239
Anesthesia duration (min)		159 (150-185)	160 (146-170)	0.378
Operation duration (min)		110 (93-145)	107 (93-122)	0.381
Tourniquet duration (min)		102 (96-119)	100 (90-108)	0.080
Desflurane dosage (Vol%)				
	Min	3.1 (3.0-4.0)	0 (0-0)	<0.001
	Max	5.0 (3.0–7.0)	0 (0-0)	<0.001
Thiopental sodium (mg)		300 (250-350)	0 (0-0)	<0.001
Propofol dosage (*μ*g/ml)				
	Min	0 (0-0)	2.5 (2.0-3.0)	<0.001
	Max	0 (0-0)	3.5 (3.0-4.5)	<0.001
Remifentanil (*μ*g)		1346 (1150-1468)	1337 (1157-1438)	0.762
Phenylephrine (*μ*g)		959 (688-1750)	945 (738-1518)	0.765
Atropine (mg)		3 (7.5%)	3 (7.5%)	1.000
Intraoperative pRBC (units)				0.358
	1	2 (5.0%)	0 (0.0%)	
	2	2 (5.0%)	2 (5.0%)	
Postoperative pRBC (units)				0.924
	1	4 (10.0%)	3 (7.5%)	
	2	3 (7.5%)	3 (7.5%)	
Postoperative bleeding (ml)		283 (215-360)	248 (170-400)	0.343

Data are expressed as mean ± standard deviations, median (interquartile ranges), or numbers of patients (%). HTN: hypertension; DM: diabetes mellitus; NOAC: non-vitamin K antagonist oral anticoagulant; pRBC: packed red blood cells.

**Table 2:** Changes of creatinine (Cr), creatinine phosphokinase (CPK), and lactate dehydrogenase (LDH).

		Desflurane group (*N* = 40)	Propofol group (*N* = 40)	*P*
Cr (mg/dl)				
	Preop	0.8 (0.6-0.9)	0.7 (0.6-0.8)	0.095
	Post 6 h	0.7 (0.6-0.9)	0.7 (0.6-0.8)	0.378
	Post 24 h	0.7 (0.6-0.8)	0.6 (0.6-0.8)	0.266
CPK (U/l)				
	Preop	95.0 (79.0-104.0)	59.5 (52.5-112.0)	0.149
	Post 6 h	130.0 (112.0-150.5)	128.5 (116.0-149.5)	0.969
	Post 24 h	215.0 (160.0-248.5)	215.0 (172.0-225.0)	0.836
LDH (IU/l)				
	Preop	477.0 (438.5-530.0)	487.0 (426.5-610.0)	0.912
	Post 6 h	544.0 (455.5-645.0)	543.0 (494.0-606.5)	0.954
	Post 24 h	440.0 (377.5-470.0)	477.5 (335.0-568.0)	0.368

Data are expressed as median (interquartile ranges). Cr: serum creatinine; CPK: creatinine phosphokinase; LDH: lactate dehydrogenase; Preop: before anesthesia induction; Post 6 h: at 6 h after discharge from postanesthesia care unit; Post 24 h: at 24 h after discharge from postanesthesia care unit.

**Table 3:** Results of arterial blood gas analysis.

		Desflurane group (*N* = 40)	Propofol group (*N* = 40)	*P*
PF ratio				
	Preop	408 (348-481)	409 (381-491)	0.400
	Before TQ	430 (355-473)	356 (282-468)	0.107
	After TQ	418 (391-481)	389 (251-459)	0.009
	Post 1 h	351 (275-414)	317 (225-459)	0.473
Hematocrit (%)				
	Preop	37 (33-38)	35 (35-37)	0.273
	Before TQ	34 (30-36)	32 (31-33)	0.076
	After TQ	33 ± 3	32 ± 2	0.379
	Post 1 h	34 (31-38)	33 (33-35)	0.737
Lactate (mmol/l)				
	Preop	0.8 (0.7-0.9)	0.9 (0.8-0.9)	0.201
	Before TQ	0.9 (0.8-1.2)	0.9 (0.7-1.4)	0.517
	After TQ	1.8 ± 0.4	1.8 ± 0.5	0.725
	Post 1 h	1.9 (1.6-2.5)	2.0 (1.4-2.7)	0.988

Data are expressed as mean ± standard deviations or medians (interquartile ranges). PF ratio: the ratio of arterial oxygen partial pressure to fractional inspired oxygen; Preop: before anesthesia induction; Before TQ: just before tourniquet deflation; After TQ: at 5 min after tourniquet deflation; Post 1 h: discharge from postanesthesia care unit.

## Data Availability

The datasets used and analyzed during the current study are available from the corresponding author on reasonable request.
